# The Alpine LGM in the boreal ice-sheets game

**DOI:** 10.1038/s41598-017-02148-7

**Published:** 2017-05-18

**Authors:** Giovanni Monegato, Giancarlo Scardia, Irka Hajdas, Francesca Rizzini, Andrea Piccin

**Affiliations:** 1 CNR - Institute of Geosciences and Earth Resources, Torino, Italy; 2 Instituto de Geociências e Ciências Exatas, Universidade Estadual Paulista, Rio Claro, Brazil; 3 Laboratory of Ion Beam Physics, ETH Zürich, Switzerland; 4 Mantova Province, Environment and Land Planning Sector, Mantova, Italy; 5 Lombardia Region, Territorial Department, Milano, Italy

## Abstract

New chronologic and stratigraphic constraints from the Garda morainic amphitheater define the extension of the last glaciation in the Adige-Sarca system and improve the Alpine LGM dataset. Together with the available chronology of the Rhine and Tagliamento systems, our results indicate a synchronous maximum culmination of Alpine glaciers during the LGM, which anticipated by about 3.5 ka the maximum extension of the Eurasian Ice Sheet (EIS). This is ascribed to the sensitivity of Alpine glaciers to the availability of moisture from southerly circulation, as recently documented by speleothem δ^18^O curve from Sieben Hengste (7 H). According to global circulation models, the waxing of the North American Ice Sheet (NAIS) at 26–23 ka pushed the North Atlantic jet stream southwards. This enhanced precipitation rates in southern Europe by advection of moisture from the Mediterranean Sea, triggering expansion of the Alpine glaciers. NAIS waning after 23 ka led to the gradual re-establishment of westerly circulation and renewal of a moisture supply to northern Europe, feeding the EIS to its maximum volume. Reduced supply of moisture from the Mediterranean Sea sealed the fate of the Alpine glaciers, which entered a final recessional phase after 22 ka and faded out after 17.5 ka.

## Introduction

In the last decade, factors controlling the spread of mountain glaciers during the Last Glacial Maximum (LGM) and their relationship with the growth of boreal ice-sheets have been debated^1, 2^. Many mountain glacier maxima^1, 3^ appear to be out of phase with the global sea-level minimum at 20.5 ka^4^, which corresponds to maximum ice-volume expansion. This incongruity raises questions about controls on Alpine glacier extension and the sensitivity of glacial response to climate cooling at the scale of a specific mountain range. From this perspective, atmospheric controls over the European Alps, such as precipitation rates and latitudinal insolation, have been extensively studied in the last several years, leading to circulation models^5–7^ that link the growth of mountain glaciers^8^ to variations in precipitation rates. In the context of intensified latitudinal pressure gradients and increased circulation strength from subtropical moisture reservoirs to mid-latitude regions^9^, the nearby Mediterranean Sea is considered to be an additional source that contributed to the growth of mountain glaciers in the Alps and in the Balkans^10^.

**Figure 1:**
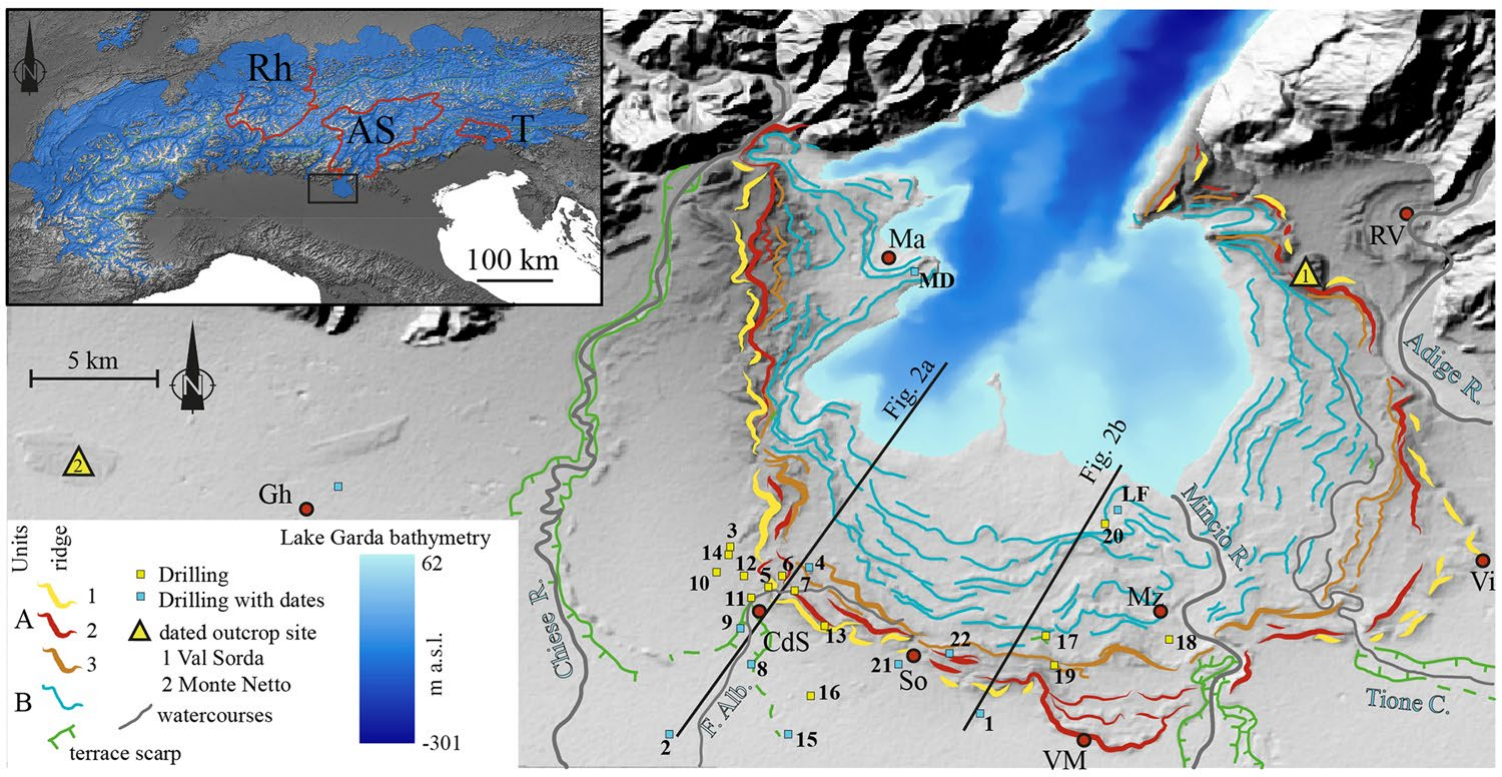
(**a**) Extension of the Alpine glaciers during the LGM^53^ and outline of the considered catchments (AS: Adige/Sarca, RH: Rhine, T: Tagliamento), study area in the black frame. (**b**) Digital Elevation Model of the Garda end-moraine system with the major geomorphologic features, drainage (*F.Alb*.: Fosso Albanella) and locations (CdS: Castiglione delle Stiviere; Gh: Ghedi; Ma: Manerba; Mz: Monzambano; So: Solferino; RV: Rivoli veronese; Vi: Villafranca; VM: Volta Mantovana) are outlined. Drillings are listes as in Table S1, LF: Lake Frassino core^37^; MD: Manerba core^26^. Maps are generated using CNR-licensed software ArcGIS 10.4 (http://www.esri.com/software/arcgis), and Adobe Illustrator CS5.1, (http://www.adobe.com/au/products/illustrator.html).

Oxygen isotope data from speleothems^8^ in the Central Alps indicate that major glacier advances had a primary moisture source from southerly advection, pointing to the Mediterranean Sea and the subtropical area as source regions for high precipitation rates in the Alps. This advection track would have affected mostly the east-central sector of the Alps^11^, facilitating the development of large glaciers even in small fore-alpine catchments, such as the Tagliamento^12^. Following this circulation model, regional differences in the timing of valley glacier advances have been linked to the location (N or S of the Alps) of the glaciers’ accumulation areas^8^. This interpretation is based on the only two glacial systems in the Alps for which firm chronologic constraints are available: the Tagliamento^12, 13^ and the Rhine^14^. However, their large difference in catchment size (9,400 km^2^ for the Rhine and 2,500 km^2^ for the Tagliamento; Fig. 1) places in doubt their suitability for generating regional models.

The present study provides new chronological and stratigraphic constraints for the Garda end-moraine system (GEMS), formed by the piedmont lobe of the Adige-Sarca glacier, which occupied the largest accumulation basin on the southern side of the Alps. The analysis of 22 cores drilled in the GEMS and in its outwash plain, coupled with 14 new radiocarbon dates, allowed construction of a robust chronology, which supports interpretation of the last glaciation in the GEMS. On the basis of this chronostratigraphic framework, we can assign the GEMS to the Alpine LGM and construct an update regional scenario for the Alpine glaciers. Comparisons of these date with speleothem isotope records and the curves of waxing and waning of boreal ice-sheets allows a more comprehensive analysis of phasing between the Alpine glaciers and the large ice-sheets of the Northern Hemisphere.

The Garda morainic amphitheater is the result of repeated glacier advances from the Adige-Sarca catchment during the Pleistocene. Its LGM accumulation area of nearly 15,000 km^2^ included many summits exceeding 3000 m above sea level (a.s.l.). Along the southern side of the Alps, the GEMS is the largest end-moraine system (630 km^2^) and, for a long time, it has been considered the classic example of a morainic amphitheater^15^. Secondary ice-streams also originated from the Adige-Sarca catchment. One filled the present-day Adige valley, forming a small amphitheater at Rivoli Veronese^16, 17^ east of the GEMS (Fig. 1), while others overflowed into Astico^18^ and Brenta valleys, respectively. The latter contributed to building the large Brenta megafan^19^, whose maximum aggradation rate (3.05 mm/yr) occurred between 27.5 to 23.6 ka, and the incision phase since ca. 17.5 ka is well documented^20^.

The extension of the LGM deposits in the GEMS has been debated since the classic Penck & Brückner study^21^ but remains unresolved^22^. Interpretations range from maximal^17, 21^ to minimal^16, 23^ extension and any chronostratigraphic assessment was mainly based on geomorphologic and pedologic considerations. A few radiometric constraints were lately obtained from different morphologic sectors of the amphitheater. A radiocarbon age of 33.7–30.7 ka cal BP^24^ from a chernozem paleosol buried by a till in the Val Sorda section (Fig. 1), subsequently supported by concordant luminescence dates^25^, gave a maximum age for the occurrence of LGM glaciation in the foreland. A last glacial advance during the recession stage is indicated by pollen analysis and a ^14^C date at 17.7–17.3 ka cal BP^26^, giving a minimum age for glacier collapse. Outside the amphitheater, the onset of outwash plain aggradation is indicated at 26.0–25.2 ka cal BP in the Ghedi RL1 core^27^ (Fig. 1) and loess deposition at Monte Netto occurred between 24.6 ± 2.9 ka and 16.24–15.77 ka cal BP^28^.

## Methods

**Figure 2:**
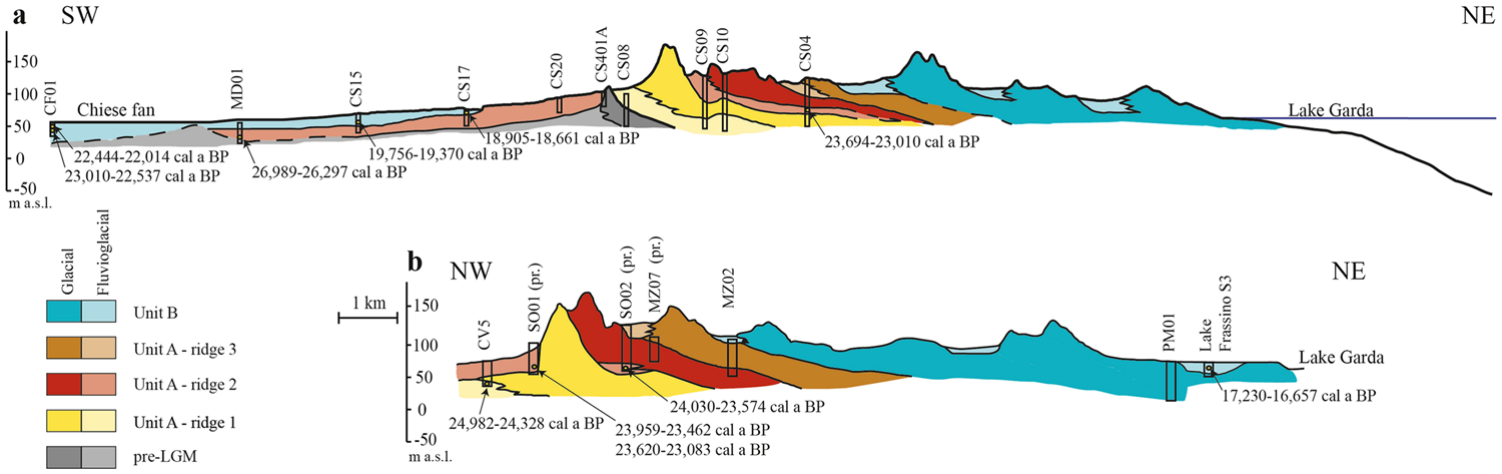
Cross-section of the Garda end-moraine system showing the stratigraphic architecture of the units related to the LGM glaciation; the labelled drillings (rectangle) and related dates (yellow spot) are referred to Table 1; the trace of the profiles is reported in Fig. 1.

Information about the analyzed sediment cores is reported in Table S1. Core description and facies analysis have been performed on the basis of sedimentary textures and structures, and their vertical variations. Lithofacies codes^29, 30^ were adopted for labeling core logs (Fig. S1). The occurrence of accessory materials, including roots, organic matter, wood fragments, bioturbation and weathering was highlighted. Cores were used for the stratigraphic interpretation of the Garda end-moraine system through cross-sections, as shown in Fig. 2.

**Table 1:** Results of AMS radiocarbon analysis.

Code	CORE	Depth (m)	Material	^14^C age (BP)	Calibrated ages (a BP) 2σ range	δ ^13^C (o/oo)
Beta-389100	CS15	−4.90	Gyttja	20,020 ± 70	24,311 to 23,860	−27.2
Beta-389099	CS15	−8.40	Plant debris	16,200 ± 50	19,756 to 19,370	−30.5
Beta-389103	CS15	−12.40	Plant debris	19,180 ± 70	23,407 to 22,930	−29.5
Beta-389104	CS17	−9.10	Plant debris	15,530 ± 50	18,905 to 18,661	−26.9
Beta-389102	CV5	−38.00	Plant debris	20,470 ± 80	24,982 to 24,328	−26.6
Beta-389105	SO01	−43.40	Plant debris	19,680 ± 70	23,959 to 23,462	−26.1
Beta-389101	SO01	−43.45	Plant debris	19,410 ± 70	23,620 to 23,083	−26.3
Beta-406507	CF01	−7.60	Plant debris	18,390 ± 60	22,444 to 22,014	−29.0
Beta-406596	CF01	−10.45	Plant debris	18,920 ± 70	23,010 to 22,537	−27.5
Beta-410008	SO02	−71.20	Plant debris	19,770 ± 60	24,030 to 23,574	−24.6
ETH-67305	MD01	−3.31	Plant debris	4,360 ± 23	5,027 to 4,857	−28.2
ETH-67306	MD01	−22.30	Plant debris	22,342 ± 73	26,989 to 26,297	−22.8
ETH-67304	CS04	−45.80	Plant debris	22,418 ± 74	27,069 to 26,425	−26.3
ETH-67307	CS04	−46.20	Plant debris	19,484 ± 52	23,694 to 23,192	−26.9
ETH-67308	CS04	−46.90	Plant debris	19,307 ± 53	23,496 to 23,010	−25.6

The proposed stratigraphic architecture and subdivision into two major units derives from the integration core interpretation and correlation (Fig. 2) with the geomorphological analysis of moraine patterns and spill pathways. The latter included analysis of a 3 arc seconds Digital Elevation Model (DEM; resolution ca. 90 m) and aerial photos, coupled with field surveys. Previous geologic maps^16, 17^ were widely used to support morphologic correlation between the morainic ridges and the terraced outlet systems. The presence of large internal outwash plains is signaled by the transition from unconfined or weakly confined sedimentation of the glacier and the outwash rivers to a strict confinement of the sedimentation. Radiocarbon ages (Table 1) were calibrated using the OxCal 4.2 and INTCAL13 calibration curve^31^. The δ ^13^C values of samples analyzed at ETH were measured on graphite^32^.

## Results

Large and complex morainic amphitheaters, such as the GEMS, are the result of long-lasting oscillations of the glacier snout, which pushes and overrides the sediments carried from the drainage catchment^30, 33–35^. Because of the latitude of the Alps, the Garda was likely a warm-based glacier with a continuous back-and-forth behavior leading to annual production of new landforms^36^. In this context, the potential for preservation of minor landforms is limited and only the products of major advances (major ridges) or recessional phases (larger inter-morainic outwash plains and lakes^33^) are preserved.

The present-day drainage network in the GEMS shows three distinct arrangements (Fig. S2) consisting of an outer radial pattern from the external moraines to the frontal outwash plain, an annular pattern in the intermediate sector formed by the major internal collectors and an inner centripetal drainage towards Lake Garda from the innermost moraines. Prior topography, sculpted in Mesozoic-Cenozoic bedrock, split the glacier snout into several small side-lobes and controlled the present configuration of Lake Garda as well (Fig. 1). The outermost moraines show the highest elevation and lateral continuity. They are organized into 10-km-wide arches (Castiglione delle Stiviere, Volta Mantovana, and Villafranca) enclosing lower, inner landforms that are arranged in largely concentric, discontinuous moraines with a spacing of 10 to 15 km, in the frontal sector. On the sides of the GEMS, moraines become closer and merge upstream, as in the Val Sorda sector (Fig. 1).

We subdivide the GEMS into two major morphologic units:

Unit A includes the outer group of moraines, which can be ascribed to the maximum advance, due to their prominent position and higher elevation. Fluvioglacial deposits are morphologically and stratigraphically connected to the frontal moraines, and indicate continuous aggradation. Unit A is subdivided into three sub-units, named “ridges” (Figs 1 and 2) because of their morphological expression, then these have been correlated with specific stratigraphic intervals in the cores (Fig. S1).

Unit B includes the innermost moraines, which are lower in elevation and spatially separated from the outer moraines by confined outwash plains. These are in morphologic continuity with the terrace staircases within the incisions of the major outlets (Fosso Albanella creek, Tione creek and Mincio River). This group records the oscillations of the glacier front, which took place during the recessional phase (Fig. 1).

The integration of information provided by landforms and available drillings allowed us to specify the stratigraphic architecture of the sedimentary units related to glacier culminations (Fig. 2), especially in the frontal portion of the system between Castiglione delle Stiviere and the Mincio River (Figs 1 and 2).

Unit A rises from the plain up to 100 m (Fig. S2) and can be separated into three main ridges (Figs 1 and 2). Stratigraphic data from cores (SO01, SO02, CS09, CS10, CV5) indicate that moraines are made of normally-consolidated, matrix-supported diamicton, interpreted as a till related to frontal pushing of the glacier snout. At the bottom of the succession, diamicton is overconsolidated and rich in striated and polished clasts, interpreted as subglacial traction till^30^. The overall thickness is up to 60 m.

Within Unit A, Ridge 1 is the outermost moraine and overlies older glacial and fluvial deposits (cores CS02 and CS402) in the western sector of GEMS, whereas in the east-central sector it forms arches (Fig. 1). In core CV5, subglacial till referred to Ridge 1 overlies fine-grained fluvial deposits with plant debris, which were deposited around 24.9–24.3 cal ka BP (for details of radiocarbon ages see Table 1). This occurrence establishes the maximum age of the arrival of the glacier at its maximum extension, in agreement with the onset of loess sedimentation at 24.6 ± 2.9 ka on Monte Netto^28^, outside the GEMS. An older age of 27.0–26.2 cal ka BP in distal fine-grained fluvial deposits (core MD01) suggests onset of fluvial aggradation in the early advance stage. A slightly younger age of 26.0–25.2 cal ka BP in the Ghedi RL1 core^27^ is referred to aggradation in the southwest sector of the outwash plain. At most locations, Ridge 2 is proximal to Ridge 1, but at Solferino and Volta Mantovana it overstepped the more distal moraines. Fluvial deposits located between Ridges 1 and 2, which document a temporary withdrawal of the front, contain organic debris deposited around 24.0 to 23.0 cal ka BP (cores SO02 and CS04, Table 1) in the GEMS and 23.9–23.1 cal ka BP (cores SO01, Table 1) in the frontal outwash plain at similar elevations (60 m a.s.l.; Fig. 2).

Ridge 3 is located in a proximal position and lacks lobate arches due to confinement within the outer moraines. The ridges of Unit A merge in the lateral sectors of the GEMS near Solferino (Fig. 1). However, the presence of fine-grained glaciofluvial deposits between subglacial till of Ridge 3 and the previous glacial deposits (cores CS04 and MZ07) again suggests oscillations of the terminus. The associated outwash developed inside the moraine system, at about 130 m a.s.l., before flowing out onto the plain.

Unit B is related to the recessional stage and consists of several discontinuous moraines separated from the outer Unit A by large internal plains at ca.120 m a.s.l. Several lakes and plains formed between the morainic ridges, producing a marked annular fluvial pattern. The outwash was confined to three major streams, entrenched within the Unit A frontal plain in the west (Fosso Albanella), central (Mincio River), and east (Tione Creek) sectors, respectively. The facies assemblage of Unit B points to a steady decrease of glacial and glaciofluvial activity, providing the first clear indication of a decreasing ice mass. Glacial retreat lowered the spillways and merged outwash flow into the Mincio River drainage. Dating of plant debris in fine-grained deposits within the Fosso Albanella outlet (cores CS15 and CS17, Table 1) indicate deposition at 19.7–19.3 cal ka BP. This age is in agreement with the entrenchment accompanying glacial retreat, which continued until 18.6 cal ka BP (CS17). In the distal plain, 10 km downstream of Castiglione, two radiocarbon ages from organic debris yielded ages of 22.2–22.0 and 22.9–22.5 cal ka BP (core CF01). These are associated with the entrenchment of the Chiese River in the west sector of the outwash plain. The most proximal moraine of Unit B, encircling the present Lake Garda shoreline, is correlative with the “Manerba culmination” at ca. 17.7–17.3 ka cal BP^26^. Wetlands remain only in the central sector, where the oldest lacustrine deposits of intermorainic Lake Frassino are dated at 17.2–16.6 ka cal BP^37^ (Fig. 2).

## Discussion

**Figure 3:**
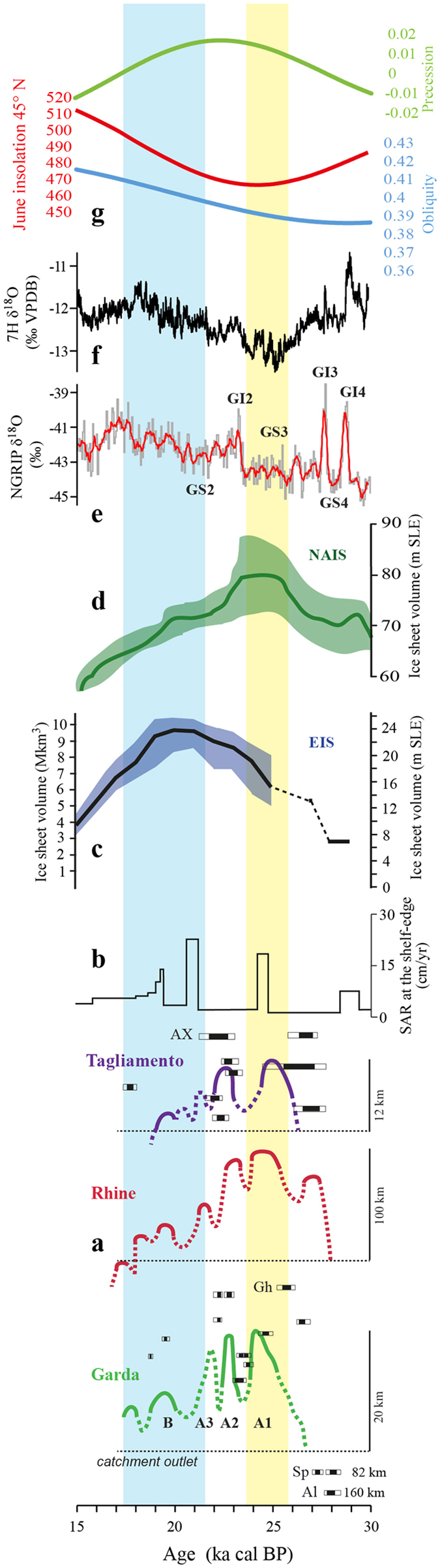
(**a**) Curves representing major culminations (yellow banner) and ice decay (pale-blue banner) of the three considered glaciers relative to the catchment outlet (dashed line) are outlined; datings for Garda catchment^27, 39, 54^ and Tagliamento^12^ are reported with 2σ range, (Al: Albeins; AX: Azzano Decimo; Gh: Ghedi; Sp: Spormaggiore). (**b**) Sediment accumulation rate in the Adriatic delta front^38^. (**c**) LGM ice volume of the North American ice sheet (NAIS)^42^. (**d**) LGM ice volume of the Eurasian ice sheet (EIS)^48^. The shaded parts of the curves c and d represent the margins of error in modelling. (**e**) NGRIP δ^18^O^55^. (**f**) 7 H δ^18^ O record from speleothem 7H^8^. (**g**) Precession (green), obliquity (blue) and June insolation at 45°N (red)^56^.

By integrating morphologic, stratigraphic and geochronological data, we can constrain most of the GEMS’ landforms to the LGM (Fig. 2), as originally proposed by Penck and Brückner^21^. The pushing of the ice snout to the frontal position took place at just after 24.9 cal ka BP, then the front had a short withdrawal that took place between 23.9 and 23.0 cal ka BP. At the same time, onset of loess sedimentation is documented from the GEMS^28^, some 30 km away. Following a second culmination, just after 23 cal ka BP, subsequent minor advances were characterized by progressive stacking of moraines related to the retreat and lowering of the ice surface, until the final collapse of the glacier occurred, around  17.7–1﻿7.3 ka cal BP^26^. This trend is mirrored in the activity of the Brenta megafan^20^, partially fed by the Adige outwash, which attained peak discharge around 27.5–23.6 ka and deactivation at 17.5 ka. New data on the Adriatic lowstand delta^38^ show a maximum sediment accumulation rate at ca. 24.6–23.8 ka (Fig. 3), in agreement with the maximum extension of the Alpine glaciers.

The dataset from GEMS, along with those available for the Rhine^14^ and the Tagliamento^12, 13^ systems, confirm that the Alpine glaciers were advancing at ca. 28 ka cal BP. At that time the Adige glacier was damming the tributary Noce Valley^39^, 82 km upstream from the valley outlet. In the northwestern Alps, the Rhine chronology matches to available data from the minor Reuss and major Rhone systems, indicating a maximum position at 24.0 ± 1.0 ka and a second large advance at 22.0 ± 1.0 ka^40^. A recessional phase, 12 km upstream, was dated at 18.6 ± 0.9 ka in the Reuss system^40^. These data point to synchronous advance of glaciers having the accumulation area totally (Reuss)^40^ or partially (Rhine and Rhone)^8, 40^ in the northern side of the Alps.

Our results confirm this synchronicity in the Alpine systems at around 25.5–24 ka (yellow banner in Fig. 3). Hence, the timing of maximum Alpine ice volume is consistent with the GS-3 stadial and well represented by the minimum in the isotopic curve of the 7 H speleothems^8^. All glacier systems show an “inner maximum” (withdrawal and a re-advance) after 23 ka, at the onset of GS-2, in phase with the second minimum in the 7 H record (Fig. 3). Oscillation in the Tagliamento system seem to have occurred earlier than elsewhere, which could be related to its small, low-elevation catchment, albeit uncertainties in chronology could also explain this apparent shift.

**Figure 4:**
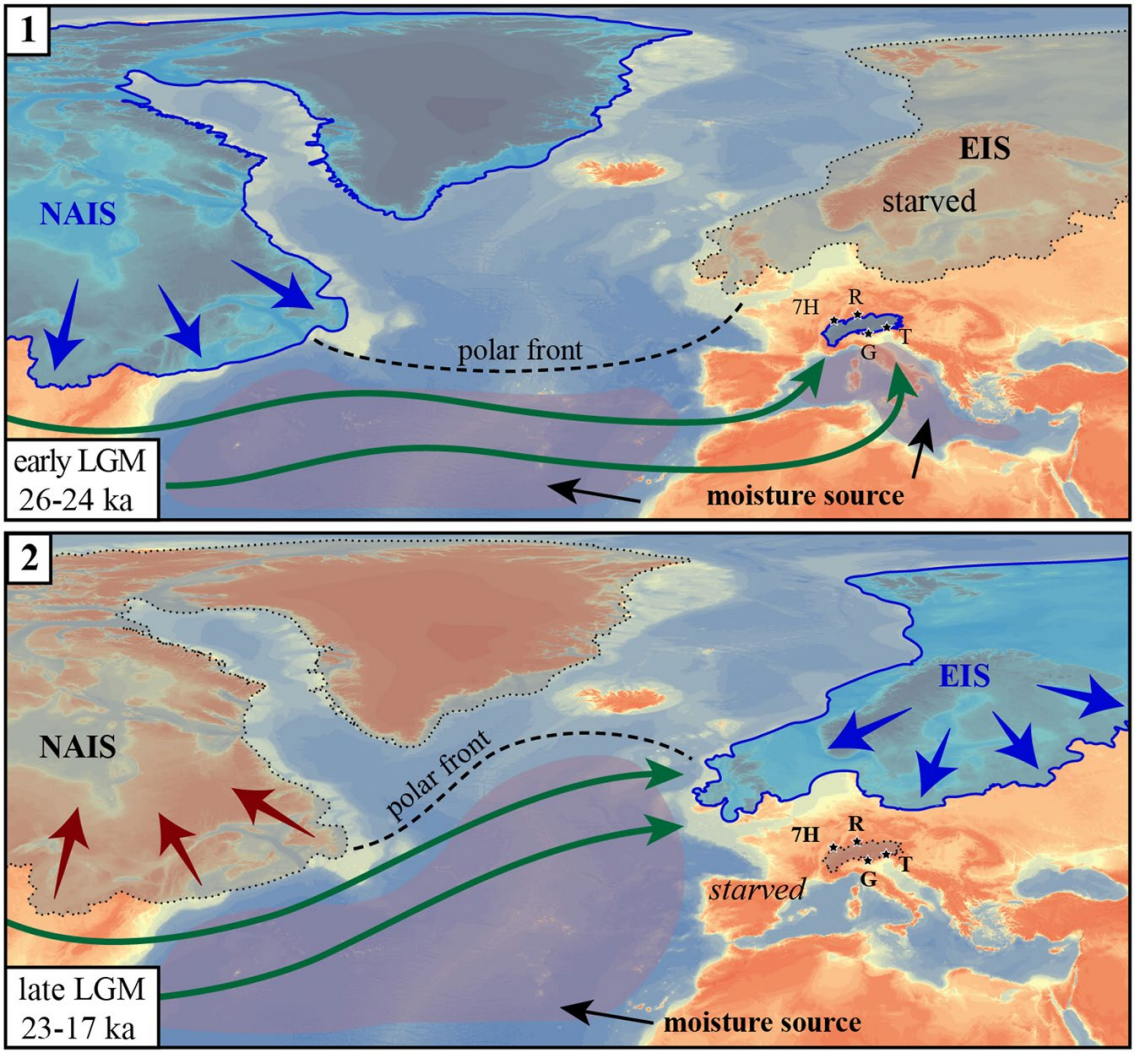
Schematic synthesis of the two phases of the LGM: (1) the topography of the NAIS produced changes in atmospheric circulation forcing the jet stream (green arrows) over southern Europe and the Mediterranean, which became an additional moisture source increasing precipitation over the Alps triggering synchronous Alpine glaciers advance; whereas the EIS waxing was slowed down. (2) The withdrawal of the NAIS at the increasing of the insolation drove the northward migration of the polar front, which move the advection towards the EIS and likely the Greenland; whereas the Alpine glaciers, underfed by precipitation, started to retreat. Maps are generated using CNR-licensed software ArcGIS 10.4 (http://www.esri.com/software/arcgis), and Adobe Illustrator CS5.1, (http://www.adobe.com/au/products/illustrator.html).

Several recessional moraines were deposited within the GEMS between 22 ka and 17.7 ka (Fig. 2), corresponding to a progressive downwasting of the glacier and convergence of the intermorainic outwash plains with the Mincio River. Similar behavior of the Tagliamento glacier is documented between 22 and 19.5 ka^13^, while the Rhine system has several recessional units spanning from 21.5 to 17.3 ka^14^, and a similar pattern was dated in the Reuss system^40^. The decline of the three glaciers corresponds with the shift of the isotopic record of 7 H speleothems towards more positive values (light blue banner in Fig. 3) and it is interpreted as a shift towards a dominant westerly trajectory of the air masses^8^. Marked lowering of the LGM ice-surface is dated to 18 ka in several major Alpine catchments^41^. The Adige-Sarca and Rhine glaciers, with large and elevated accumulation areas in the axial sector of the Alps, maintained extensions adjacent to the foreland. Conversely, the smaller and less elevated Tagliamento glacier faded away rapidly, suggesting that this kind of system had a rapid feedback to sharp climate variations.

The chronostratigraphic comparison glaciated systems on both sides of the Alps supports synchronous ice build-up during the LGM without significant regional differences in the timing of major valley glacier advances.

In summary, the synchronous build-up of mountain glaciers in the Alps occurred after the obliquity minimum, and had its climax at around 26–24 ka BP (Fig. 3), in phase with the final waxing of the North American Ice Sheet^42^ (NAIS). According to atmospheric circulation models^7, 43, 44^, the topographic anomaly created by the thick NAIS^45^ led to the southward shift of the polar front and the North Atlantic jet stream towards the latitude of the Iberian Peninsula. This drove the developing advection tracks from the Mediterranean Sea towards the Alps and Central Europe (Fig. 4). Because of this configuration, the change in moisture source from the North Atlantic to the Mediterranean Sea fed the Alpine region with moisture from the south, facilitating the rapid growth of Alpine glaciers. Oxygen isotope data from 7 H^8^﻿ well mark the continuation of advection from the Mediterranean Sea until the GS2, at around 22 ka, when a gradual shift toward more positive values occurred due to re-establishment of westerly circulation^8^.

The synchronous build-up of mountain glaciers in the Alps was, apparently, out of phase with development of the last Eurasian Ice Sheet (EIS, Fig. 3). The maximum extent^46–48^ of the EIS had a time-transgressive migration from west to east. It reached its maximum volume at around 21 ka^48^, after the insolation minimum (Fig. 3), when Alpine glaciers and the NAIS^49^ were already in a recessional stage. Also, the Greenland Ice Sheet had its maximum extension between 24–17 ka^50^. According to atmospheric circulation models, a thick NAIS may have prevented a more massive build-up of the EIS^43^. This may explain why the EIS reached its climax after the GS-2, when the NAIS was losing volume and reclaiming the jet stream at higher latitudes (Fig. 4). An earlier maximum for the Scandinavian sector of the EIS at the end of MIS 3 has been constrained by OSL chronology^48, 51^ and, perhaps, the change in circulation due to the waxing NAIS may have just slowed down the EIS growth. At least, no evidence for a MIS3 early advance in the Alps has been reported for the Alps thus far.

We argue that, in a general context of low insolation values during the global LGM, a change in moisture sources had a major impact on the development of mountain glaciers and ice sheets on the European continent. The phasing of volume climax patterns between NAIS and Alpine glaciers confirms that the NAIS played a major role in atmospheric circulation, and consequent moisture distribution, as suggested by previous models^8, 11, 43, 44^. In addition to these primary controls, the extent of sea-ice cover at the Heinrich Stadial 2^52^ may also have contributed to reduction in moisture supply from the North Atlantic to the EIS. The correlation between the shift from negative to more positive values in the 7H isotope record and the obliquity trend, established by speleothems data^8^, suggests an ultimate connection between the obliquity, the development of a thick NAIS, and moisture availability, at least over continental Europe. From this perspective, the Alpine LGM, anticipating the EIS maximum, can be considered a consequence of the change in atmospheric circulation caused by the larger NAIS. This suggests that major ice-advances in the Alps during the Pleistocene cold stages may have a similar cause-effect trigger.

In conclusion, our reconstruction of Alpine glacial dynamics points to a distinct climatic shift during the LGM, which controlled the sedimentary evolution of the morainic complexes both in mountain and in ice-sheet environments. Such a difference between an “early” LGM (26–23 ka) and a ‘late’ LGM (23–17 ka) should be taken into account in future circulation and climatic models.

## Electronic supplementary material

Supplementary Information
