# Supplementary material for: The Alpine LGM in the boreal ice-sheets game
Source: Sci Rep. 2017 May 18;7:2078. doi: 10.1038/s41598-017-02148-7 (PMC5437061; doi:10.1038/s41598-017-02148-7)
Supplement: Supplementary file 1 — Supplementary Information [file 41598_2017_2148_MOESM1_ESM.pdf]

The Alpine LGM in the boreal ice-sheets game

Giovanni Monegato[1,*], Giancarlo Scardia[2], Irka Hajdas[3], Francesca Rizzini[4], Andrea Piccin[5]

[1]CNR - Institute of Geosciences and Earth Resources, Torino, Italy

[2]Instituto de Geociências e Ciências Exatas, Universidade Estadual Paulista, Rio Claro, Brazil

[3]Laboratory of Ion Beam Physics, ETH Zürich, Switzerland

[4]Mantova Province, Environment and Land Planning Sector, Mantova, Italy

[5]Lombardia Region, Territorial Department, Milano, Italy

* corresponding author: g.monegato@csg.to.cnr.it

Supplementary information

Table S1. Location and depth of the drillings

	Code	Locality	WGM84_GMS_E	WGM84_GMS_N	Elevation (m a.s.l.)	Depth (m)
1	CV05	Cavriana	10°36'01.7158"	45°20'10.7350"	76	40
2	Cf01	Castel Goffredo	10°26'33.0700"	45°19'14.6100"	57	20
3	CS02	Castiglione delle Stiviere	10°28'44.5958"	45°24'43.0166"	117	65
4	CS04	Castiglione delle Stiviere	10°30'58.0695"	45°24'23.6423"	121	75
5	CS08	Castiglione delle Stiviere	10°29'18.8992"	45°23'31.7900"	100	50
6	CS09	Castiglione delle Stiviere	10°30'00.9018"	45°24'09.3171"	130	84
7	CS10	Castiglione delle Stiviere	10°30'29.9328"	45°23'42.8614"	113	60
8	CS15	Castiglione delle Stiviere	10°29'07.3976"	45°21'31.0261"	70	30
9	CS17	Castiglione delle Stiviere	10°29'05.8364"	45°22'24.0260"	77	25
10	CS19	Castiglione delle Stiviere	10°27'42.2161"	45°23'27.0638"	93	40
11	CS20	Castiglione delle Stiviere	10°28'24.2764"	45°23'12.4092"	92	21
12	CS401a	Castiglione delle Stiviere	10°28'48.9759"	45°24'00.1919"	108	30
13	CS402	Castiglione delle Stiviere	10°30'08.9775"	45°23'12.0490"	132	57
14	CS407	Castiglione delle Stiviere	10°28'44.5939"	45°24'43.1997"	117	30
15	MD01	Medole	10°30'26.9662"	45°19'15.7681"	57	30
16	MD03	Medole	10°31'59.6243"	45°20'49.3502"	83	40
17	MZ02	Monzambano	10°38'04.3838"	45°22'29.0969"	107	54

18	MZ03	Monzambano	10°41'09.0769"	45°22'06.3825"	91	54
19	MZ07	Monzambano	10°39'20.8226"	45°21'25.0672"	111	31
20	PM01	Ponti sul Mincio	10°39'49.6515"	45°25'38.6061"	86	51
21	SO01	Solferino	10°33'39.8268"	45°21'28.6995"	106	51
22	SO02	Solferino	10°34'25.8587"	45°21'56.2933"	129	73

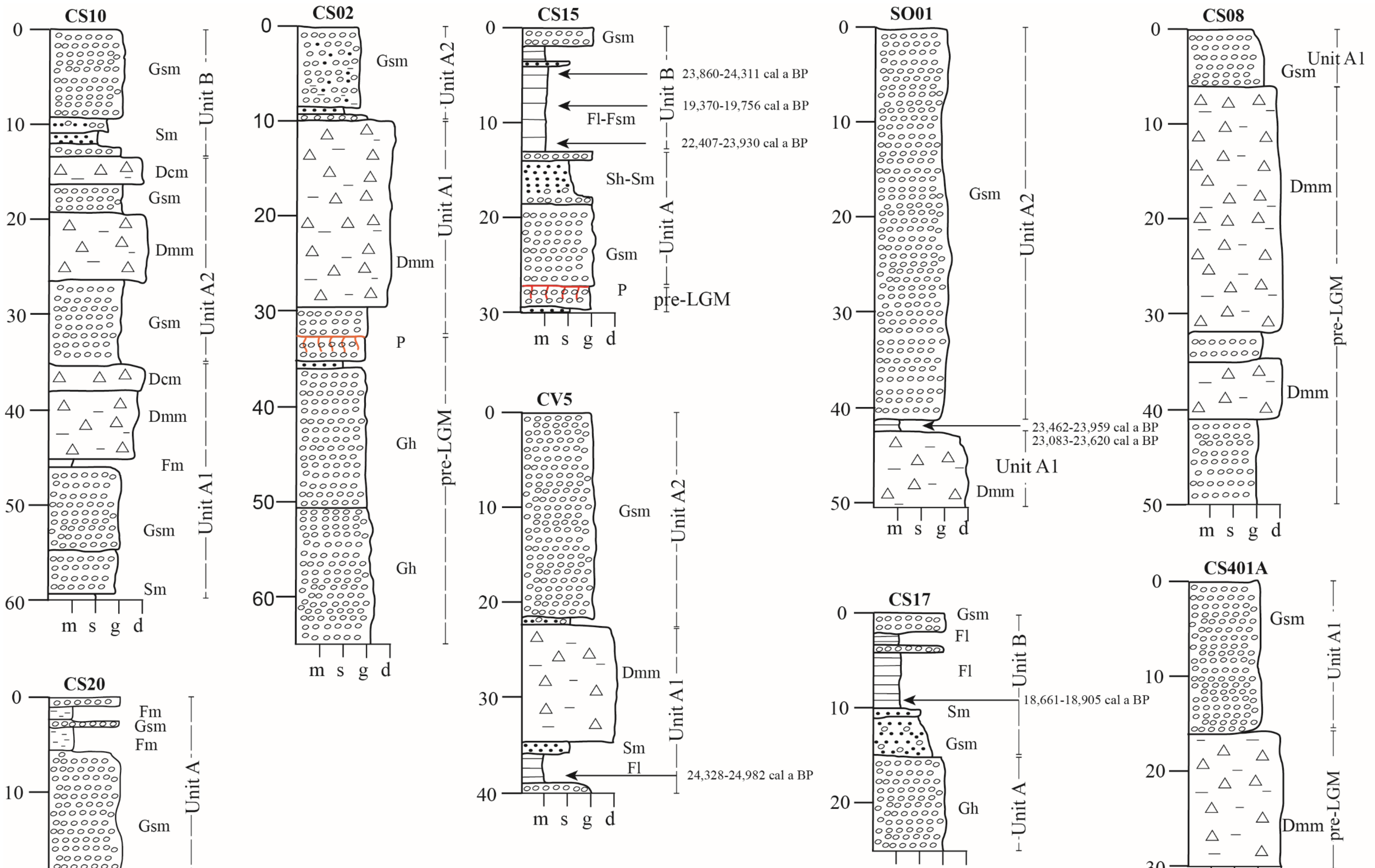

Figure S1_a
The Alpine LGM in the boreal ice-sheets game
Giovanni Monegato1,*, Giancarlo Scardia2, Irka Hajdas3, Francesca Rizzini4, Andrea Piccin5

Fig. S1 (a-b)- Log of the boreholes drilled in the southwestern sector of the Garda end-moraine system (location reported in Tab. 1s and Figs. 1 and 2)

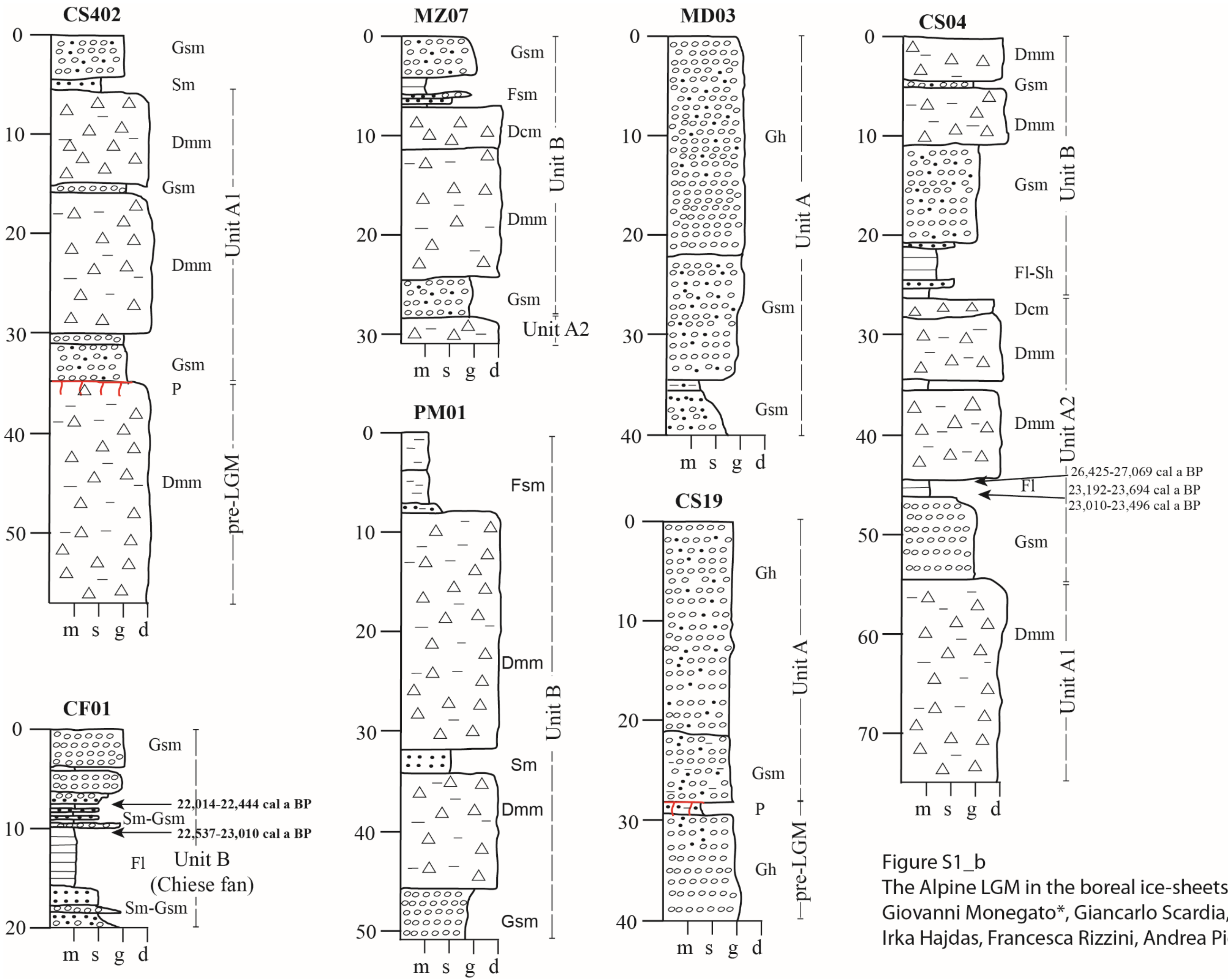

Figure S1_b
The Alpine LGM in the boreal ice-sheets game
Giovanni Monegato*, Giancarlo Scardia,
Irka Hajdas, Francesca Rizzini, Andrea Piccin

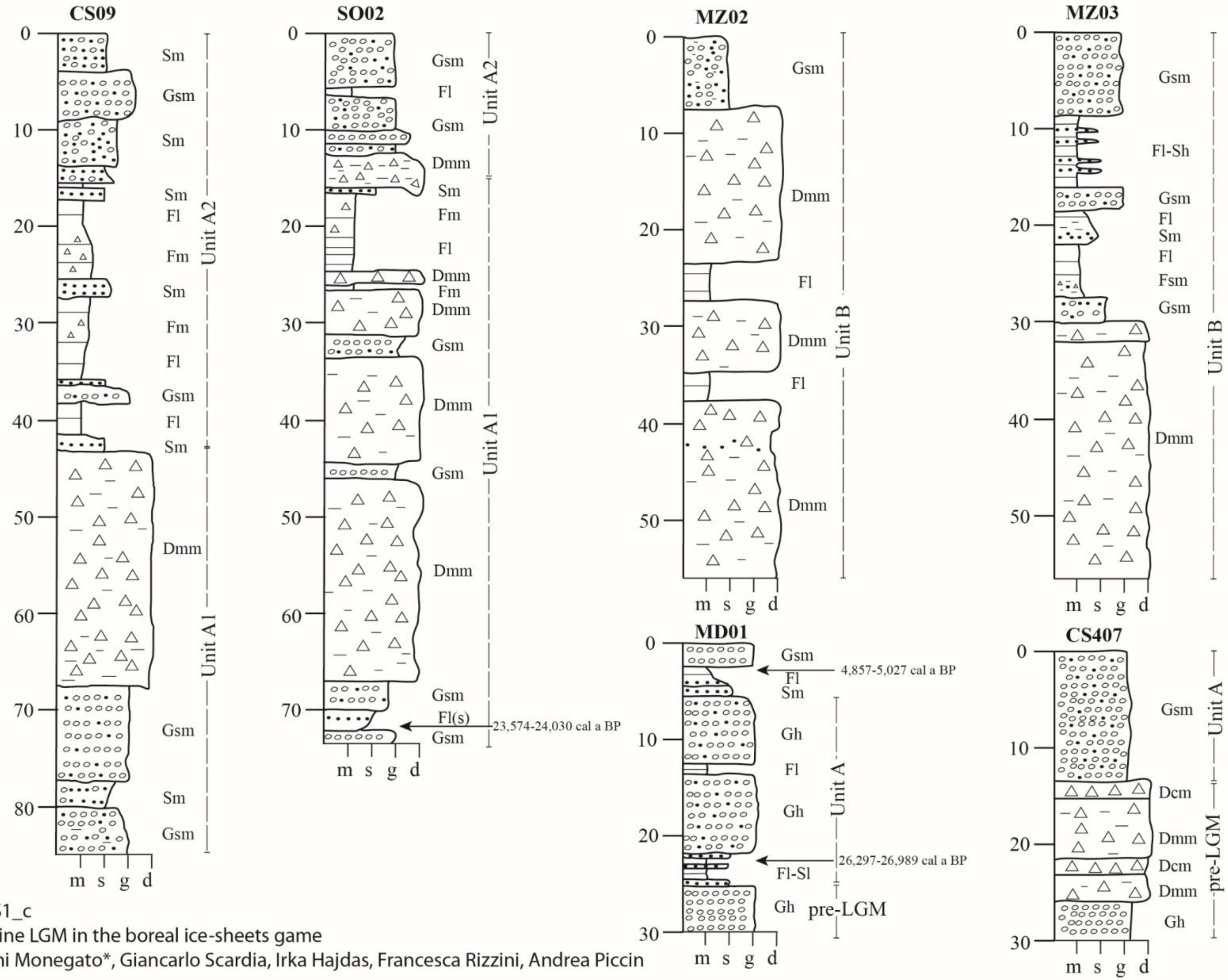

Figure S1_c
The Alpine LGM in the boreal ice-sheets game
Giovanni Monegato*, Giancarlo Scardia, Irka Hajdas, Francesca Rizzini, Andrea Piccin

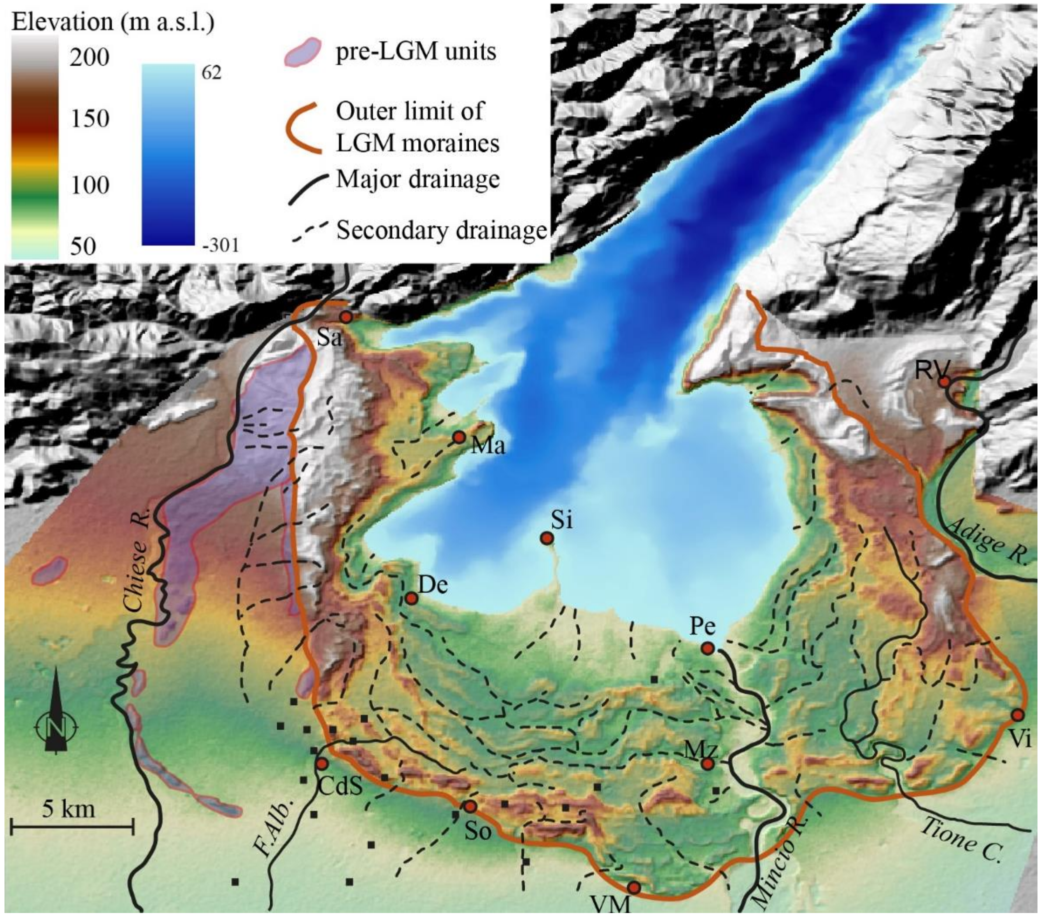

Fig. S2 - Digital Elevation Model of the Garda end-moraine system with outlined the drainage patterns and the extent of the pre-LGM moraines. (F.Alb.: Fosso Albanella Creek; CdS: Castiglione delle Stiviere; Ma: Manerba; Mz: Monzambano; Pe: Peschiera; Si: Sirmione; So: Solferino; RV: Rivoli veronese; Vi: Villafranca; VM: Volta Mantovana).

Maps are generated using CNR-licensed software ArcGIS 10.4 (http:// http://www.esri.com/software/arcgis), and Adobe Illustrator CS5.1, (http://www.adobe. com/au/products/illustrator.html).
